# Diurnal evolution of urban tree temperature at a city scale

**DOI:** 10.1038/s41598-021-89972-0

**Published:** 2021-05-18

**Authors:** Thuy Trang Vo, Leiqiu Hu

**Affiliations:** Department of Atmospheric and Earth Science, The University of Alabama in Huntsville, Huntsville, AL USA

**Keywords:** Ecology, Plant sciences, Climate sciences, Ecology, Environmental sciences

## Abstract

Despite the importance of urban trees’ surface temperature in assessing micro-climate interactions between trees and the surrounding environment, their diurnal evolution has been largely understudied at a city-wide scale due to a lack of effective thermal observations. By downscaling ECOSTRESS land surface temperature imaginary over New York City, we provide the first diurnal analysis of city-scale canopy temperature. Research reveals a remarkable spatial variation of the canopy temperature during daytime up to 5.6 K (standard deviation, STD), while the nighttime STD remains low at 1.7 K. Further, our analysis shows that the greenspace coverage and distance to bluespaces play an important role in cooling the local canopy during daytime, explaining 25.0–41.1% of daytime spatial variation of canopy temperatures while surrounding buildings modulate canopy temperature asymmetrically diurnally: reduced daytime warming and reduced nocturnal cooling. Built on space-borne observations and a flexible yet robust statistical method, our research design can be easily transferable to explore urban trees’ response to local climate across cities, highlighting the potentials of advancing the science and technologies for urban forest management.

## Introduction

The rapid expansion of built-up areas within metropolitan cities inevitably elevates urban temperatures, which is commonly known as the Urban Heat Island (UHI) effect^1^. Plantation of urban trees is considered as one of the widely applied heat mitigation strategies in cities^2^. In fact, urban trees are the most observable component of green infrastructure within mosaic urban settings^3^ and offer considerable aesthetic value, ecological services^3^, and cooling benefits to reduce heat stress and consequently summer energy demands^4,5^. Trees face various stresses from urban effects such as high heat load from surrounding paved grounds and buildings^6–8^, limited water availability, and poor soil conditions^9,10^. As a consequence, urban environment can largely weaken the trees’ health and functionality^3^.

Canopy temperature, commonly described by radiant temperature measured by thermal sensors (or Land Surface Temperature (LST)), is a resultant of surface energy balance between leaves and the ambient atmosphere. The tree canopy temperature is primarily governed by the tree’s canopy architecture (leaf density, canopy shape, etc.), meteorological conditions (ambient temperature, air humidity, etc.) and plant physiology^11^. Knowledge of the canopy temperature in urban environment is critical to understand the micro-climate influences on trees’ health^12–15^ and how this delicate ecosystem burdens trees’ living conditions. For example, the stress-induced reduction in evapotranspiration (ET) rate considerably hinders plant growth and development and directly reduces evapotranspirative cooling^16,17^, resulting in an increased canopy temperature^18^.

The canopy temperature, its relationship with evapotranspiration, and the influence on the ambient environment have been extensively studied at the leaf and canopy scale^19–24^. Different from the natural ecosystem, the inherent heterogeneity of the urban ecosystem can largely differentiate the growth and health conditions of trees as well as their interactions with the micro- to local-scale climatic environments. Yet, little is known about the spatial and temporal variation of the canopy temperature and their responses to the urban landscape structure at the city scale. For example, a previous local-scale study unveiled that trees are warmer over the impervious dominated surface than vegetative dominant area both daytime and nighttime^22^. Interception of upward long-wave radiation over human-made surfaces having higher surface temperatures consequently results in higher canopy temperature, which partly explaining this temperature discrepancy. However, the urban environment interaction at a city-wide scale is much more complicated than that at a local scale, as urban landscape vary across the city. For example, urban greenspaces consist of various forms, such as gardens, parks, lawns, and street trees are featured with many diverse spatial structures across the city^25^. Largely limited by the spatial coverage of local thermal observations, whether piecemeal fine-scale findings are generalizable to a greater domain in the built environment remains unclear.

Besides the impacts of ground materials (e.g., greenspace and impervious materials)^21,22^, other local environmental factors (e.g., bluespaces, urban morphology) can modulate the canopy temperature through different physical processes. However, their effects have not been studied yet, particularly from observational evidence. For example, buildings cast shadows on trees, directly reducing the heating of tree canopies during the daytime while constraining the evapotranspiration due to reduced direct solar radiation^17^. Building clusters reduce the wind turbulence and trap the long-wave radiation within the urban “canyons” due to a low sky view factor^19,26^, possibly slowing the cooling of tree canopies at night^27^. Furthermore, nature or man-made waterbodies (thereafter, bluespaces), often co-exist with greenspaces in urban landscapes^28^, and strongly influence air temperature patterns in cities^29^. Daytime cooling and possible nighttime warming of bluespaces on nearshore areas have been documented in urban climate studies^30–32^. Even though these studies do not directly investigate the canopy temperature, such contrasting diurnal effects on ambient air temperature can further alter the canopy temperature at the watersides^22^. In addition, recent local-scale studies discovered a nonlinear effect of tree coverage on ambient air temperature^31,33,34^ and many studies documented the strong negative relationship between urban LST and vegetation coverage^35,36^. Even though, it is still unclear how urban canopy temperature responds to tree/vegetation coverage, surrounding buildings, bluespaces diurnally, due to lack of spatially representative observations.

Overall, the diurnal evolution of canopy temperature remains largely understudied at a city scale, and it requires further investigation of how the local built environment modulates the tree temperature diurnally in order to effectively inform the urban forestry management and to potentially maximize their ecological benefits and heat mitigation during hot summers. Observations from space are often beneficial for large-scale and routine surveys. However, the majority of moderate-resolution thermal sensors are on-board sun-synchronous orbiters, largely limiting the diurnal representation of temperature observations. Moreover, most space-borne thermal imaginary ($\sim$ 100 m or coarser spatial resolution) do not resolve temperatures at the tree or tree-cluster scale. Thus, it becomes challenging to study canopy temperature over heterogeneous urban environments with distinct temperatures of various urban elements, from natural greenspaces, bluespaces to various human-made surfaces, such as roofs and roads^35,37^. Here, we used the LST product from The ECOsystem Spaceborne Thermal Radiometer Experiment on Space Station (ECOSTRESS) mission in a precessing orbit^38^ that enables moderate spatial resolution ($\sim$ 70 m) observations at various times of day. Further, we developed a practical downscaled scheme to estimate the canopy and other primary urban elements’ temperatures at the sub-pixel level. We aim to close the aforementioned knowledge gaps and seek to answer: (1) How does city-wide tree canopy temperature vary diurnally? (2) What environmental factors and to what extent they contribute to its spatiotemporal variation? For the first time, we provide the explicit diurnal maps of urban canopy temperature and a systematic assessment of the concomitant effects of urban greenspace coverage, urban bluespaces (distance to bluespaces), and urban morphology (building height) on shaping the urban canopy temperature pattern diurnally. 12 downscaled clear-sky ECOSTRESS LST imagery over the metropolitan area of New York City (NYC) represent a distinct time of day during the peak growing season, allowing us to assess a detailed diurnal course of canopy temperature variability (approximately every 2 h). To reduce the impact of daily weather variation on LST acquired at different days, spatial anomalies of canopy temperature ($\Delta LST_{Canopy}$) were used for diurnal pattern analysis, which were estimated by subtracting the domain mean of LST at each overpass from the corresponding canopy temperature map. Generalized Additive Models (GAMs)^39^ were used to statistically evaluate the relative roles of local environmental factors on influencing tree canopy temperatures across the city.

## Results

### Diurnal evolution of canopy temperature at the city scale

**Figure 1:**
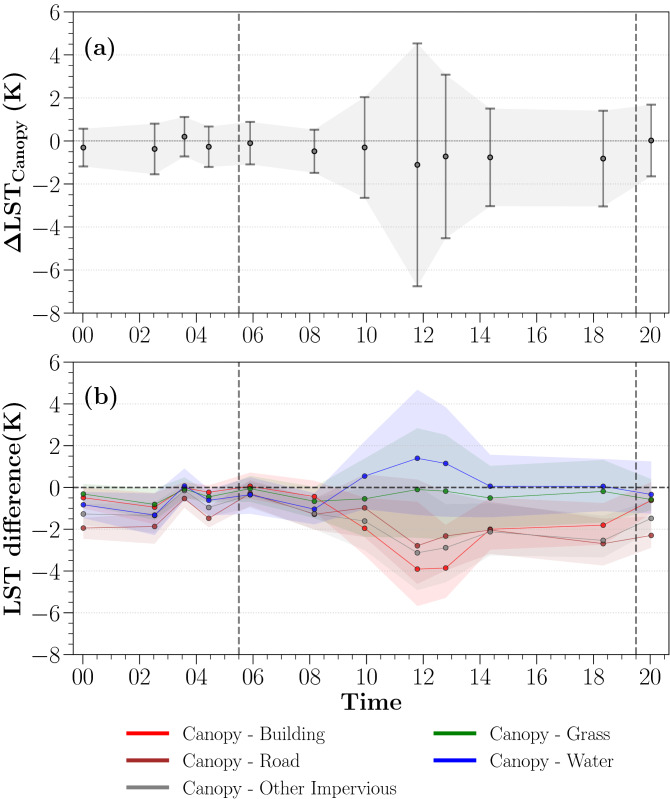
Diurnal evolution of the canopy temperature. (**a**) The spatial mean (dots) and STD (bars) of $\Delta LST_{Canopy}$ summarized at the city scale, (**b**) the mean and STD of temperature difference between tree canopies and other urban elements at the pixel level. Vertical dashed lines indicate the sunrise and sunset at the local time.

Canopy temperature exhibits a substantially diurnal difference in spatial variation in the summer and early autumn months (June–October) (see Supplementary Table S1 for a general overview of selected ECOSTRESS imaginary). Spatial variation of daytime canopy temperature is up to five times higher than that of nighttime variation (Fig. 1a). For example, the daytime spatial STD of $\Delta LST_{Canopy}$ reaches to 5.6 K at noon when it coincides with the time of peak LST in the diurnal cycle^40,41^. From the late night to early morning (20–8 Local Time (LT)), the spatial variation remains relatively stable with the STD of around 1.7 K. This diurnal canopy temperature generally follows the land-cover LST variation, suggesting a strong local effect on modulating the canopy temperature across the city.

The mean spatial anomalies of canopy temperature reaches its absolute maximum of 1.2 K at noon in the diurnal cycle (Fig. 1a) (12 LT). Due to a rise in latent heat partitioning over tree canopy as increasing in temperature and solar radiation throughout the morning, the temperature discrepancy between the canopy temperature and the domain mean LST (the spatial mean LST of the whole city) increases from 0.4 K (10 LT) up to the peak at noon. The contrast of mean canopy temperature gradually declines after the peak at noon and trees remain slightly cooler by 0.8 K than the domain before the sunset (13–18 LT). The nocturnal canopy temperature is comparable to the domain mean LST with less than 0.4 K difference (20–10 LT).

Further, we summarized the mean and STD of temperature differences between tree canopies and their surrounding urban elements locally (Fig. 1b). Generally, tree canopies are persistently cooler than ambient urban elements throughout the day, except bluespaces. The human-made surfaces, including buildings, roads and other impervious areas (e.g., railways, parking lots, etc.), have the largest contrast to the canopy temperature during the day (10–18 LT). The buildings are about 3.9 K warmer than the tree canopies on average at noon, followed by other impervious areas (3.1 K) and roads (2.8 K) when the diurnal LST reaches its daily maximum. Tree canopies have relatively greater resilience to temperature increase than surrounding man-made surfaces during daytime. Similar to daytime patterns, tree canopies remain cooler than man-made surfaces at night, but at much weaker magnitudes ($\sim$ 0.9 K cooler on average; specifically, 0.4 K for buildings, 1.0 K for other impervious areas and 1.4 K for roads) (20–08 LT) (Fig. 1b). No strong diurnal variation between tree and grass surface temperatures is found, although the grassland is still warmer than the ambient tree canopies on average 0.5 K throughout the day, partly attributed to the closer contact to the ground surface and typically a weaker ET rate^11^. On average, tree canopies are found warmer than the bluespaces around noon (10–13 LT) from 0.5–1.4 K with large spatial variations. At night, an opposite trend suggests that bluespaces are warmer than tree canopies by up to 1.3 K (2 LT). Overall, we found that the daytime contrast between tree canopy and other urban element surface temperatures is higher compared to the nighttime contrast; yet, these differences are diverse among human-made and natural urban elements diurnally.

### Spatiotemporal patterns of canopy temperature

**Figure 2:**
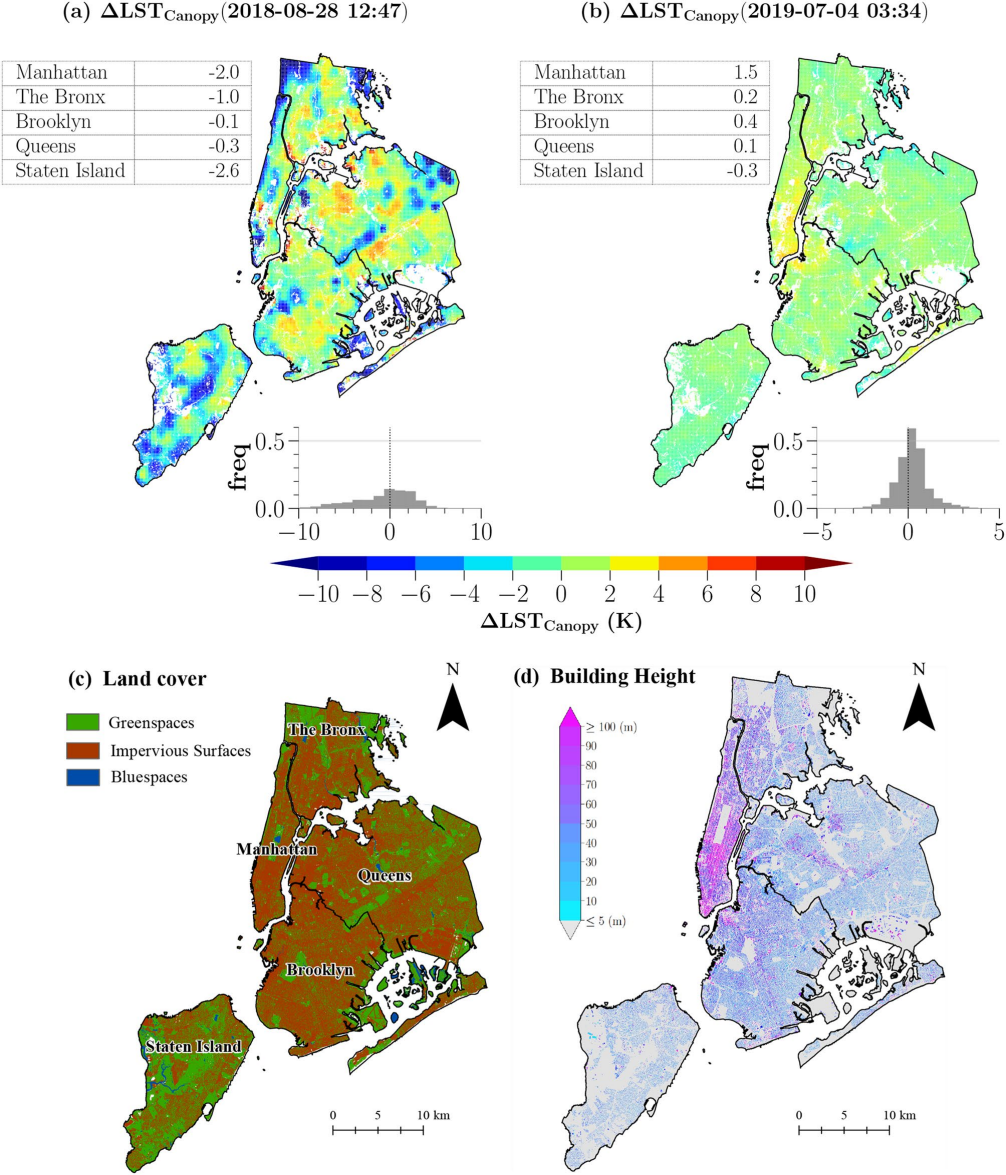
Spatial anomalies of canopy temperature ($\Delta LST_{Canopy}$) for day (**a**) and night (**b**). The histograms show the distribution of $\Delta LST_{Canopy}$. Borough mean of $\Delta LST_{Canopy}$ are shown in the table (K). Summary of urban land covers (**c**) and building height distribution (**d**) of NYC. Summary of urban land covers (**c**) and building height distribution (**d**) of NYC. Maps created in Python 3, using the canopy temperature dataset generated based on the downscaled approach (a and b) and in ArcGIS 10, using NYC Land Cover 2017^42^ and Building vector dataset^43^ (**c**, **d**) (links are provided in the “Data availability” section).

We present examples of day and night maps of $\Delta LST_{Canopy}$ over the whole NYC in Fig. 2a,b. Cooler canopy temperature is linked with greater greenery coverage, particularly during the daytime. For example, parks (e.g., central parks in Staten Island and Manhattan) (Fig. 2c) are cooler than trees at highly sealed areas (e.g., residential areas in Brooklyn and Queens) up to 16.5 K during daytime (Fig. 2a). The cooler canopy temperature is also observed along the watersides during the daytime. Both patterns show correlations between the canopy temperature with the tree coverage and locations with respect to water shorelines, which emphasize evidence of cooling effects of urban greenspace and bluespaces on the tree canopy temperature during the daytime.

Nocturnal warmer canopy temperature is observed in most boroughs, except for Staten Island (Fig. 2b). Trees in Manhattan at night are about 1.5 K warmer relative to the domain mean LST, especially at Lower Manhattan and the Upper East Side where dense high-rise buildings are clustered (Fig. 2d). Such a canopy temperature pattern suggests a critical role of urban morphology on the canopy temperature during nighttime.

### Canopy temperature modulated by major local environmental factors

To understand the complex impacts of urban environment on the spatiotemporal variability of canopy temperature, we incorporated the major known environmental factors in the GAMs model analysis, namely, greenspaces (green area fraction, GAF), bluespaces (distance to waterbodies, DTW), and surrounding building morphological characteristics (building height, BH). GAMs are a flexible approach allowing the quantification of non-linear controls of confounding factors on $\Delta LST_{Canopy}$ spatially and diurnally^31,44^. Due to the common coexistence of various greenery, e.g., trees, shrubs, and grassland, in the urban landscape and their similar diurnal temperature patterns (Fig. 1b), meaningfully distinguishing various types of greenspaces in statistical models is challenging (see Supplementary Figure S1 for further explanation of coexistence of these greenery in NYC). Thus, we accounted for influences from the total greenspace coverage on canopy temperature rather than the tree coverage alone.

**Figure 3:**
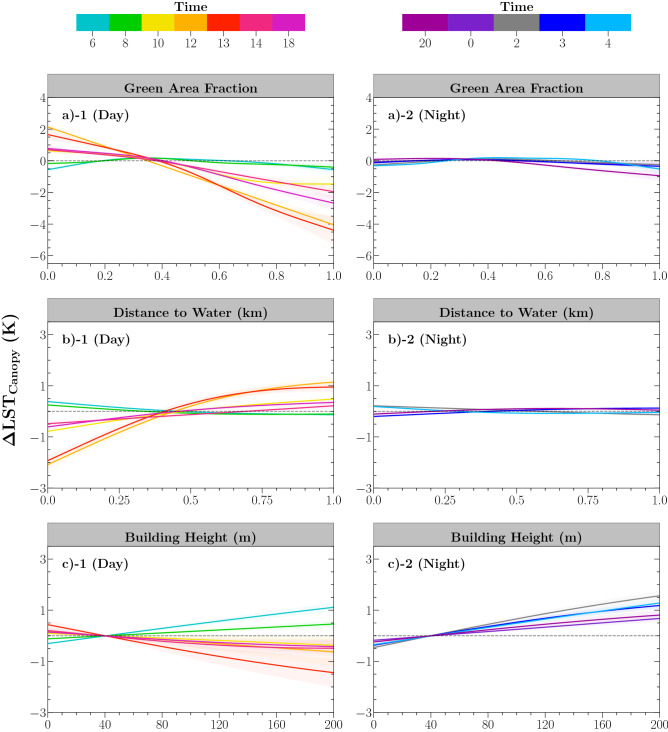
Estimated effects of urban greenspaces, distance to waterbodies, and building height on the spatial and diurnal patterns of $\Delta LST_{Canopy}$; the left column is for daytime hours and the right column is for nighttime hours. Domain mean LST was used as a reference value for $\Delta LST_{Canopy}$. $\Delta LST_{Canopy}$ equals zero is indicated as black dashed lines. The shaded regions illustrate the confidence interval 95% of GAMs fitting.

**Figure 4:**
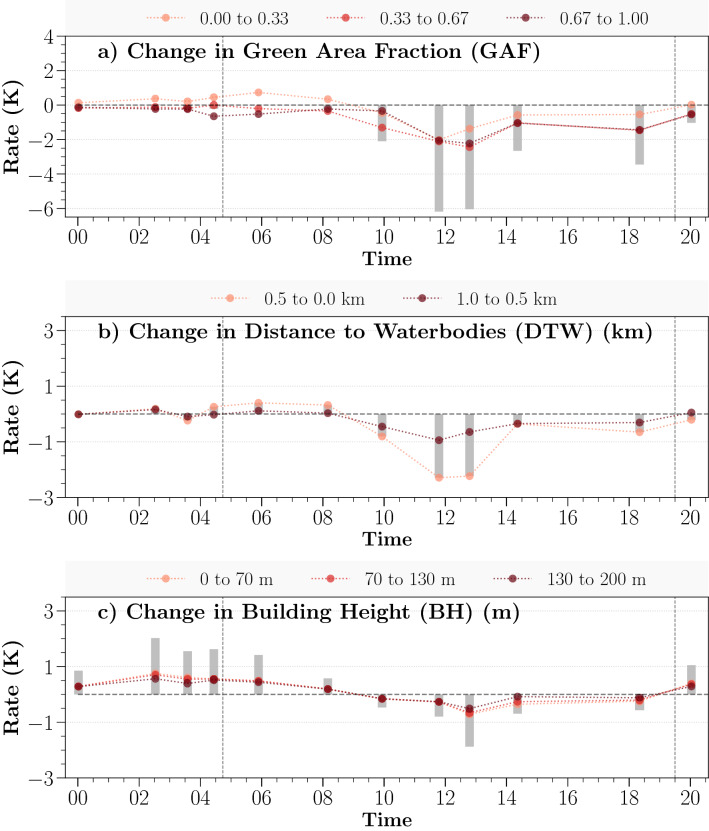
The diurnal change of canopy temperature controlled by three studied urban environmental factors: (**a**) for GAF, (**b**) for DTW, and (**c**) for BH. The colors distinguish the change in independent variable. The bars depict a possible total temperature effect from each selected variable of GAMs fittings, negative value for cooling and positive value for warming effect.

#### Influences of urban greenspace coverage

Previous findings revealed the enhanced cooling effects of greater greenspace coverage on urban ambient temperature^31,33,34^. A similar relationship is observed between GAF and the canopy temperature diurnally, showing a greater greenspace coverage associated with a cooler canopy temperature (Fig. 3a-1,a-2). The daytime impact of GAF is strong particularly at noon when LST is highest, which contributes to 2.3–6.0 K (10–18 LT) of daytime spatial variation of the canopy temperature (Fig. 4a). Unlike the strong daytime effect, the nocturnal canopy temperature is less sensitive to the GAF change (Fig. 3a-2).

#### Influences of urban bluespaces on nearshore trees

A clear signal of the daytime cooling effect from urban bluespaces is unveiled in Fig. 3b-1. The cooling benefit on waterside trees can extend up to about 0.5 km from the shore diurnally. Unsurprisingly, the effect non-linearly declines as increasing distance away from shorelines. For example, the canopy temperature is about 0.5–2.0 K cooler within 0.5 km away from the shorelines during daytime hours (Fig. 4b), which is almost twice as much as the magnitude of trees located further than 0.5 km. The land-water temperature difference induces breezes that bring cooler air inland^30^ meanwhile trees have a reduced heat and water stress which further promotes the ET during the daytime^45^, jointly leading to noticeable cooler canopy temperature at the watersides^46^. In contrast, warmer nocturnal bluespaces elevate the tree canopy temperature nearshore, but the impact is much weaker in a range of 0.0–0.6 K (Fig. 3b-2).

#### Influences of surrounding buildings

Shadows cast by tall buildings cool surrounding trees effectively during the daytime (0.5–2.0 K), evident in Fig. 3c-1, accounting for 17.0% of total daytime spatial variation of canopy temperature (Fig. 4a–c). Around noon, trees in tall building districts remain cooler than those in low-rise residential areas, partly due to the reduced direct sunlight during the daytime hours in the deep street canyons. The building height effect, however, is much weaker compared to GAF (2.3–6.0 K (41.1%)) and DTW (0.7–3.0 K (25.0%)).

Urban geometry also plays a significant role on governing nocturnal canopy temperature (e.g., up to 1.5 K at 2 LT) (Fig. 3c-2). Opposite to its daytime effect, the presence of tall building clusters reduces the tree canopies cooling due to smaller sky view factor^19^, leading to a warmer canopy temperature at night compared to those tree canopies at low-rise residential areas.

### Canopy temperature assessment by boroughs

**Figure 5:**
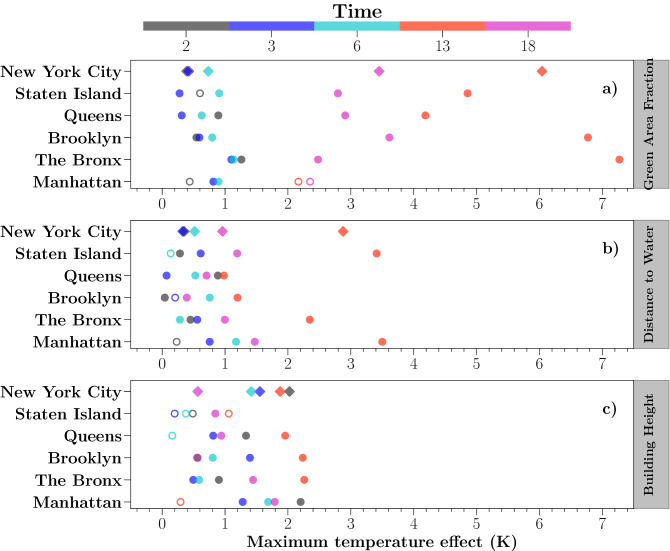
Estimated maximum temperature effect (MTE) effects on the canopy temperature at the borough level from GAF (**a**), DTW (**b**) and BH (**c**). The circle symbol represents the effects of each borough. The diamond symbol represents the city-scale results. The filled symbols indicate a statistical significance estimation in GAMs ($p < 0.05$). The colors suggest different time of day. MTE was calculated based on the difference of maximum and minimum predicted $\Delta LST_{Canopy}$ from the GAMs. Due to the reason that the distribution of buildings height is diverse among boroughs, different ranges of buildings height were selected from each borough: Manhattan: 0–400 m; The Bronx: 0–100 m; Brooklyn: 0–100 m; Queens: 0–100 m; and Staten Island: 0–70 m.

There are five boroughs in NYC, characterized by distinguishable urban architecture and greenspace distribution^47^ (see Fig. 2c). For instance, Manhattan is mostly well-known for its dense skyscrapers while Staten Island has the greenest neighborhoods. To further understand the joint effects on the canopy temperature with various urban types, a summary of borough-level GAMs analysis at selected times is presented in Fig. 5, showing the potential maximum effect of each factor on canopy temperature (MTE) estimated from GAMs (see Supplementary Figure S3 to S7 for detailed GAMs fittings at the borough level).

While the GAF and DTW effects are consistent among boroughs agreeing with the city-scale result, the effect of BH is much more diverse which emphasizes the complicated effect of urban morphology on controlling the canopy temperature at the local scale. GAF shows a similar cooling role in modulating the canopy temperature diurnally (Fig. 5a) but different in terms of the magnitude among boroughs. More specifically, the MTE reaches up to 7.2 K in The Bronx and followed by Brooklyn for 6.8 K at noon (13 LT). Nocturnal MTE controlled by GAF is much weaker in the range of 0.25–1.25 K. Strong daytime bluespace effects are found in Staten Island and Manhattan, which are mostly surrounded by rivers and open waters (Fig. 5b). Nocturnal effects of bluespaces are weaker as found in the city-scale result. The effect of building height at the borough level is much diverse. Manhattan shows the most apparent nighttime warming on the canopy temperature by up to 2.2 K (Fig. 5c). Oppositely, in The Bronx, Brooklyn, and Queens, strongest daytime cooling effect from tall buildings is about 2.3 K, 2.3 K and 2.0 K, respectively, contrasting with a much weaker nighttime effect. Staten Island shows the least morphological impact on the canopy temperature due to dominant low-rise buildings in this region (see Fig. 2d). In general, the daytime GAF effects among all boroughs as well as for the whole of NYC are strongest, which points out the most predominant daytime cooling effects caused by cluster size effect of greenspace, followed by the effects of urban bluespaces and urban morphological effects.

## Discussion and conclusions

There is an increasing need of improving management of urban forests across metropolitan areas worldwide. With this study, we examine tree temperature diurnal behavior in this highly modified environment. Our study provides the first overview of diurnal evolution of canopy temperature at a city scale. We found that the daytime spatial variation of the canopy temperature is much higher than nighttime variation. The finding underlines the great sensitivity of the daytime canopy temperature to the surrounding environment, which coincides with diurnal patterns of LST^31,40^. This research has found a greater impact of urban greenspaces compared to urban bluespaces and building height on modulating daytime canopy temperature. Bluespaces also benefit for tree development, however, limited to the nearshore regions. We highlight the asymmetric diurnal effects of surrounding tall buildings on the canopy temperature: enhanced daytime cooling from the shadow effects and nocturnal reduced cooling due to small sky view factor within deep street canyons.

Our results have shown a strong daytime spatial variation of the canopy temperature anomalies while a weak nocturnal variation. Knowledge of tree canopy temperature could improve the understanding of heat exchange between trees and the urban environment. It is clear that during the day, trees respond sensitively to surrounding urban structures (e.g., land cover distribution and morphology) evidently by the existence of various hot and cold canopy temperature spots across the city. For instance, in the central urban park of Staten Island (e.g., Latourette Park), trees are 6 K cooler than the domain mean and appear to be a ‘cool island’ in the center of this borough. On the other hand, hot spots of the canopy temperature are found at dense low-rise residential neighborhoods in Brooklyn and Queens, which are up to 4 K warmer than the domain mean. Besides, trees are found to be cooled down along coastlines in Staten Island due to the influences of urban bluespaces (e.g., Raritan Bay and Lower Bay). The nighttime coupling between ambient and tree temperature is evidently strong. Similar to previous studies on tree coverage on air temperature^34,48^, canopy temperature shows a negative non-linear relationship with increasing greenspace coverage. Besides, despite the strong daytime effect of GAF being observed on canopy temperature, we found the relationship is near linear. Sea breezes influence the air temperature pattern in NYC^49,50^, showing that southeasterly coastal winds during the afternoon hours might reduce overall UHI across the city. We found that such a strong cooling effect of bluespaces on trees not further than 0.5 km from shorelines, which might partly explained due to effects of sea breeze cooling down nearshore trees during daytime hours. Dominant roles of human-built features (e.g., sky view factor, building height^48^) associated with an enhanced UHI-related effect in characterizing nocturnal air temperature patterns in NYC has been found in the previous study^48^. Our finding shows the noticeable nighttime effect of building height on canopy temperature proven by the warmer canopy temperature in densely built areas. We have directly linked the urban morphological features to explain the nocturnal canopy temperature. It is commonly known that higher nocturnal UHI is possibly due to increased heat storage^51^ inside urban cores. High rise districts in Manhattan have higher thermal inertia compared to other low-rise buildings in the Bronx or Brooklyn. The stored heat from those tall buildings during daytime is thus released back to the environment at night, which causes a temperature difference between Manhattan and other regions in NYC.

To improve management of urban forests, alleviating stress of urban vegetation is a priority to sustain its benefit in urban living environment^52^. We thus emphasize the importance of mapping tree temperature patterns that can facilitate urban forestry offices to identify the stressed trees, and respond to them timely. Instinctively, trees have mechanisms to adapt against thermal stress from surrounding environment by changing their physiological functionality e.g., photosynthesis, stomatal regulation^53^. Under the effect of urbanization, trees tolerate hotter environments but at the same time it weakens their physiological functionality due to direct exposure to heat stress over a long period. As a result, trees become more vulnerable to diseases and pests in this delicate micro-climate condition^54^. Our approach thus provides a practical scheme to evaluate how urbanization affects urban trees’ health conditions and helps urban planners to minimize trade-offs or synergies between urban densification and greenspaces.

For densely built-up urban areas, greening effort by upgrading these areas with greenery is a promising key for a future sustainable and livable city^55^. Retrofitting existing buildings with greenery e.g., green facades, green roofs^56^ is also an ideal solution to optimize the benefits from greenspaces across these urbanized districts. Besides, it is vital to supply trees within those areas with appropriate irrigation to balance reduced moisture caused by urban effects, particularly for those tend to be exposed frequently to hotter built-up environment (e.g., tall building districts). Also, green infrastructure could be combined with blue infrastructure (e.g., green corridors along the rivers, rain gardens etc) to benefit from the bluespace’s cooling effect and thus to reduce heat stress on trees.

For inland cities with a lack of direct benefits from dense river networks, strategic plantation of urban trees should be carefully taken into account. It might be challenging to plant many trees across those cities and keep them alive given a condition of dominant impervious surface^57^, as their living conditions become harsher if they are further away from the natural ecosystem^58^. For isolated trees or small patches of urban trees, this negative effect can only be resolved by permanent increased irrigation to maintain their health and to increase their cooling benefits. Strategic plantation of urban trees should instead aim for increasing the cluster size of tree to enhance their resilience to warmer environment. Greater greenspace clusters improve the ambient air temperature^31,33^ and simultaneously reduce heat stress on the urban vegetation.

Different from the natural ecosystem, trees’ functionality in a complex urban environment are much more complicated as heterogeneity of the urban ecosystem can alter their growth and health conditions. Our downscaled canopy temperature dataset thus provides a moderate-resolution and reliable information at a city-wide scale to assess trees’ diurnal behaviors in such complex urban areas. Previous local-scale studies using thermal imaging^22^ or UAV^21^ to capture spatiotemporal patterns of the canopy temperature and it has been found that trees responded to urban landscapes at a local-scale, showing the tree temperature discrepancy between trees over dominated impervious and vegetative areas. Up-scaling to a city-wide scale, we unveil various aspects that could jointly influence tree temperature that are not limited to only landscape configurations. Besides, as ECOSTRESS observations can capture 90% of the continental United States (U.S.) and other major international cities in a repeat cycle of 3–5 days, we emphasize the potential of our approach in investigating the urban canopy temperature diurnal behavior in various urban forms across climate regions. Aside from protecting existing trees, another key priority to improve management of urban forests is to ameliorate tree selection across climate regions. Enhanced understanding of tree temperature diurnal behavior across climate regions might advance current knowledge of tree selection based on local climate conditions, as many tree plantings fail due to the wrong decision on tree selection^52^. Also, our moderate-resolution canopy temperature dataset could be considered as a piece of reliable information supporting complex simulations of urban landscapes and surrounding ambient conditions. Micro-climate effects of urban vegetation were commonly investigated via in situ measurement of leaf temperature at leaf or canopy level^19–24^. Acquiring such information is often challenging particularly in heterogeneous urban landscapes. One of alternative approaches to overcome this difficulty is using microclimate models^12^ e.g., the vegetation model of ENVI-met^59^. Our moderate-resolution canopy temperature dataset could provide a reliable information that possibly oversimplified leaf temperature in such simulations.

There are some caveats to this study. Firstly, we addressed some typical perspectives of urban structures on the canopy temperature in cities. As canopy temperature patterns in cities are influenced interactively by other perspectives (e.g., tree species, tree heights, etc.) (see Supplementary Figure S8 for $R^{2}$ of the GAMs), further investigations coupling the process-based models will be beneficial to physically decompose the governing processes in the urban settings. Secondly, our statistical-based approach based on spatial anomalies of urban canopy temperature spatially aggregates these patterns across the domain, and thus the results should be interpreted carefully at different scale as some uncertainties have been found by comparing city-scale and borough-based-scale studies. Finally, we constructed the pseudo-diurnal course of the canopy temperature from observations on different days. Yet, to eliminate uncertainties from daily weather variation in constructing this diurnal course, we used the canopy temperature anomalies as a reference to avoid the daily weather variation among those days. Despite the limitations, we believe that our study provides a transferable downscaled scheme of urban tree temperature at a citywide scale that would benefit the future development of urban forest management by utilizing our approach for other cities worldwide.

## Methods

### Study area

The study chose NYC, which is the largest metropolis in the U.S. with a population of over 8 million^60^. NYC comprises five boroughs (The Bronx, Queens, Brooklyn, Manhattan, Staten Island (see Fig. 2c), four of them with over one million population (except for Staten Island) symbolize the most urbanized areas in the U.S. By statistics, the city covers 783.1 ${\mathrm{km}}^{2}$ (as defined by borough boundaries, Fig. 2c) with 27.8% of natural greenspace coverage (tree canopies and grassland) and 42.4% of impervious surface (all man-made surface) (according to NYC Land Cover Map 2017^42^). Since 2007, NYC initiated the Million TreesNYC program which is targeting planting millions of new trees in 10 years as an effort of harnessing cooling benefits of urban greenspaces in the city^61^.

### Data and methods

#### Data

Clear-sky ECOSTRESS Level 2—LST imagery from summer and early autumn months (June–October) during 2018 and 2020 were used, consisting of LST and emissivity ($\varepsilon$) products^62^. The ECOSTRESS thermal sensor onboard International Space Station (ISS) offers moderate-spatial-resolution ($\sim 70$ m) and frequent thermal observations (repeating every 3–5 days at different time of day depending upon the latitude), allowing us to explicitly explore the temperature patterns at the different times of the day. In total, 12 images acquired at different times of day were identified. More details of selected images can be found in Supplementary Table S1.

Very high-spatial-resolution land cover data at 0.15 m produced by the New York City Department of Information Technology and Telecommunication (NYC DoITT)^42^ was used. Eight land cover categories, then, were aggregated into three primary classes, and summarized as the fraction at $\sim 70$ m grid that matches with the footprint of thermal observations, including greenspace fraction (tree canopies and grass/shrubs), impervious fraction (building/roof, road, other impervious and railways) and water fraction (see Fig. 2c). We used the 2015 building vector data (more than 1 million records of buildings) from NYC DoITT^43^ to characterize the local urban morphology. The area-weighted mean building height was estimated at 70 m scale, similar to the scale of fishnet summarized for fraction of land cover as mentioned above. For the GAMs analysis, the area-weighted mean building height was estimated at 350 m scale to match with the scale of fishnet used in GAMs. Similarly, the Euclidean distance to the nearest bluespaces (including all natural and man-made bluespaces greater than $500\, {\mathrm{m}}^{2}$) was calculated for all locations of trees at each $350\, {\mathrm{m}}$ grid.

#### Canopy temperature estimation

To estimate the canopy temperature at the sub-pixel level, a multiple linear regression (MLR) method was used to statistically decompose the sub-pixel temperatures among different urban elements at each pixel. Eight urban surface types were considered based on the land cover dataset, including tree canopy, grassland, and other major human-made surfaces, such as building roofs, roads, and railways. We hypothesized that the temperature of each urban surface element is similar within a smaller domain ($350\, {\mathrm{m}} \times 350\, {\mathrm{m}}$), and each urban element has a distinct surface temperature. The pixel-level thermal radiation can be described as an areal weighted sum of thermal radiation emitted from all surface elements within a given pixel in Eq. (1).

1 $$\begin{aligned} \varepsilon _{(i,j)} LST_{(i,j)}^{4} = \sum _{k=1}^{8} \varepsilon _{k(i,j)} * f_{k(i,j)} * LST_{k(i,j)}^{4} + r_{(i,j)} \end{aligned}$$

where $\varepsilon _{k}$ is the emissivity of the urban element *k*; $f_{k}$ is the areal fraction of the urban element *k* in a given pixel; $LST_{k}$ is the LST of corresponding urban element *k* in a given pixel; and *r* is the residuals.

To solve the eight unknown $LST_{k}$, a moving window with a size of 5 by 5 pixels was selected, which was balanced with the efficiency and accuracy under solutions derived from the MLR method as described in Eq. (1). A sensitivity test was implemented to decide which moving size is most appropriate for canopy temperature mapping. As conclusion, a kernel size of 5 by 5 pixels was chosen to map the spatial patterns of canopy temperature (See Supplementary Figure S9 for the sensitivity test of different kernel sizes). The MLR model fitting and parameter estimation were conducted using optimized Least-squares estimation with a constrained rule based on Trust Region Reflective (TRF) algorithm^63^. TRF algorithm was applied to reduce the impact of outliers^63^ and was adapted for a linear least-squares problem by setting constraints on the output variables^63^. A constrained rule was set based on the maximum (upper constrain) and minimum (lower constrain) LSTs in the domain for each ECOSTRESS image. Model performance of the proposed canopy and urban element temperature estimation is provided more in detail in Supplementary Materials Figure S10. In the least-squares estimation, we solved the LST for each urban element ($T_{k}$) using Eq. (1) using the following optimization problem:

2 $$\begin{aligned} \frac{1}{2} \sum _{i=1}^{n} (\varphi (\varepsilon _{k};f_{k};LST_{k}^{4}) - LST_{i,j}^{4}*\varepsilon _{i,j})^{2} \longrightarrow \min _{\mathbf {T}} \end{aligned}$$

where $\varphi (\varepsilon _{k};f_{k};LST_{k}^{4})$ is the multiple linear function.

Due to the limitation of the ISS orbit, the LSTs from different times were obtained from various days within the two year window therefore LST can be sensitive to daily weather conditions. To overcome such impact from daily temperature fluctuation, we calculated $\Delta LST_{Canopy}$ (hereafter, spatial anomalies of canopy temperature) [Eq. (3)] which was primarily used for the analysis. The spatial mean LST of the whole city $\overline{LST_{ECOSTRESS}}$ (so-called, domain mean LST) was used as a reference to estimate the spatial anomaly to provide information about the relative cooling/warming degree of urban trees compared to the domain thermal condition. To avoid the strong thermal influences of water bodies, the $\overline{LST_{ECOSTRESS}}$ only consider the land pixels. Our results primarily relied on the temperature changes in space and time, thus despite the diurnal cycle being non-synchronous, the results are still valid to reflect the spatiotemporal patterns of tree canopy temperatures.

3 $$\begin{aligned} \Delta LST_{Canopy}(i,j) = LST_{Canopy}(i,j)- \overline{LST_{ECOSTRESS}} \end{aligned}$$

#### Generalized additive models (GAMs) analysis

We used GAMs model to analyze the contributing roles of GAF, DTW, and BH to the spatial patterns of canopy temperature in R package *mgcv*^44^. The power of GAMs lies in the flexibility to define the dependence specification of the response variable on the independent variables, and it is possible to avoid the circumstance of overestimated relationship due to strong spatial auto-correlation^33^. To account for the influences of the identified environmental factors on canopy temperature, we conducted GAMs analysis at the scale of $350 \times 350\, {\mathrm{m}}^{2}$ (5 by 5 pixels) at a neighborhood scale. This scale also captures the majority of park sizes, which can better account for the clustering effects of urban greenspaces on the canopy temperature.

Fractions of GAF, DTW, and BH were defined as smooth terms in the model. The interaction term was fit using a tensor product interaction (ti) term. Here we considered the interaction terms for: (1) greenspace coverage and distance to waterbodies and (2) distance to waterbodies and building height. The degrees of freedom were restricted for each term below the *mgcv* default (six basic functions for GAF and DTW and three basic functions for BH), in order to generalize the trends of GAMs fitting curves). Spatial coordinates (x,y) were also included as a smooth term with restricted ten basic functions for the city-scale study and two basic functions for the borough-level study. The numbers of basic functions were chosen for the spatial coordinate smooth term based on the fact that the study was more interested in the large scale trends (according to smaller basic functions) of each environmental factors on the canopy temperature rather than the local-scale (according to larger basic functions). We conducted this approach for each time of day separately and the estimated effect of individual variables are illustrated in Fig. 3a–c.

## Supplementary information

Supplementary Informations.

## Data Availability

The canopy temperature dataset generated from 12 ECOSTRESS imaginary using our downscaled approach are available in the ZENODO repository, https://doi.org/10.5281/zenodo.4321389. A Python script for the downscaled approach will be available upon request. The supporting dataset (e.g., NYC Land Cover Map 2017 and NYC Building vector data) area publicly provided by NYC DoITT. The software used to generate the maps and figures are ArcGIS 10 and Python 3.
