# Supplementary material for: Diurnal evolution of urban tree temperature at a city scale
Source: Sci Rep. 2021 May 18;11:10491. doi: 10.1038/s41598-021-89972-0 (PMC8131592; doi:10.1038/s41598-021-89972-0)
Supplement: Supplementary file 1 — Supplementary Informations. [file 41598_2021_89972_MOESM1_ESM.pdf]

Supplementary Material

Diurnal Evolution of Urban Tree Temperature at a City Scale

Thuy Trang Vo and Leiqiu Hu

Table section

Table S1. Summary of ECOSTRESS LST imaginary

Date	Time (in EDT)	Domain mean LST (K)
2019-07-28T00-01-02	0:01:02	295.551
2019-09-21T02-31-50	2:31:50	288.679
2019-07-04T03-34-56	3:34:56	294.930
2019-07-01T04-26-20	4:26:20	290.945
2019-07-13T05-53-53	5:53:53	292.688
2018-08-24T08-09-44	8:09:44	293.148
2018-09-04T09-56-02	9:56:02	305.363
2019-06-28T11-47-32	11:47:32	310.979
2018-08-28T12-47-26	12:47:26	314.822
2019-10-05T14-21-33	14:21:33	295.345
2019-07-26T18-20-34	18:20:34	303.346
2019-09-20T20-02-07	20:02:07	291.698

Figure section

1. **Explanation of combining tree and grassland coverage as greenspace coverage**

To explore the statistical coexistence of vegetation types (e.g. tree canopies and grassland), we resampled the original grid size (70 m x 70 m) to larger grid size (490 x 490 m) to explore the spatial patterns of vegetation. A preliminary test was conducted to decide that the grid size of 490 x 490 m (equals to a neighbor square) is sufficient to investigate the interaction between tree canopies and grassland at a city-scale size.

The relationship between the fraction of canopy coverage and its own surface temperature is shown (Figure S1). It is clear that at fraction of canopy coverage less than 15 % is dominated by grass coverage (mean coverage fraction is 19 % for the whole domain). Within this fraction range, tree temperature is decreased linearly at the degree of 2 K per 5 % of canopy coverage. On the other hand, at a fraction of canopy coverage larger than 15 %, tree temperature remains almost constant. Therefore, we found that it is difficult to distinguish them statistically as shown that grass coverage plays a statistically important role in this model.

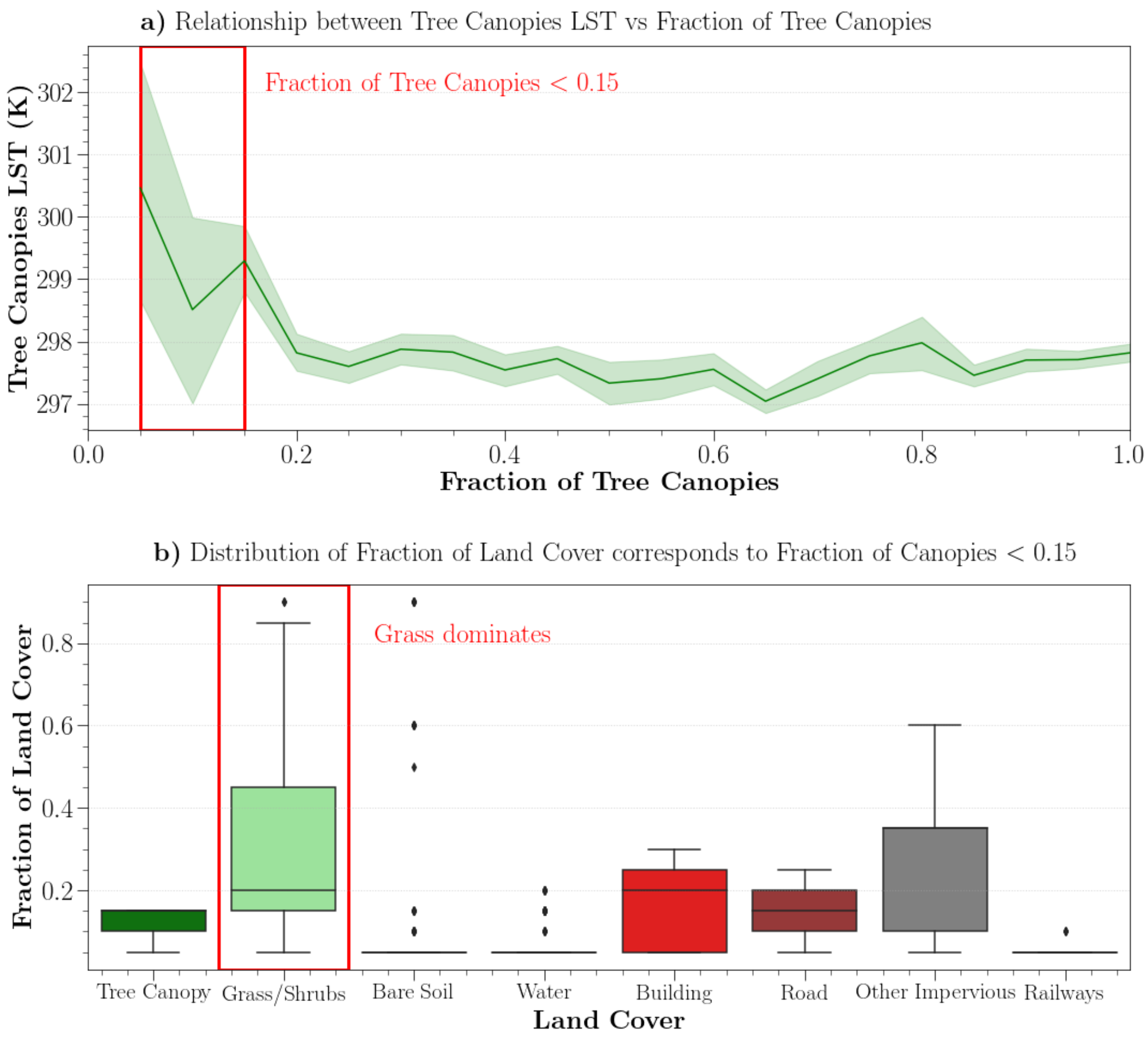

Figure S1. Coexistence effect of the tree and grassland fraction on the estimated tree LST using LST and Tree Canopy Temperature Estimation approach. Figure S1 (a) represents the linear relationship between estimated tree canopies LST and the fraction of tree canopies. The red box indicates the abnormal tree LST in the range of tree canopies fraction less than 15 %. Figure S1 (b) represents the distribution of other land cover components within this fraction range (tree canopies < 15 %). The light green box plot demonstrates the distribution of grassland, which is dominant (accounts for 20 % on average in total) compared to other land cover types.

2. Explanation of warming effects of urban bluespaces at night (3 LT observation)

On July 04 2019 (3 LT), there was an abnormal cooling Sea Surface Temperature (SST) pattern in the ocean near NYC. This pattern cools down the surface temperature of bluespaces

surrounding this city and thus cools down the nearshore tree canopies accordingly. It could be observed on July 01 2019 (4 LT) as well as other nocturnal observations that the surface temperature of water bodies is much warmer at night compared to the patterns on July 04 2019 (3 LT). These warmer patterns warmed up the nearshore tree canopies.

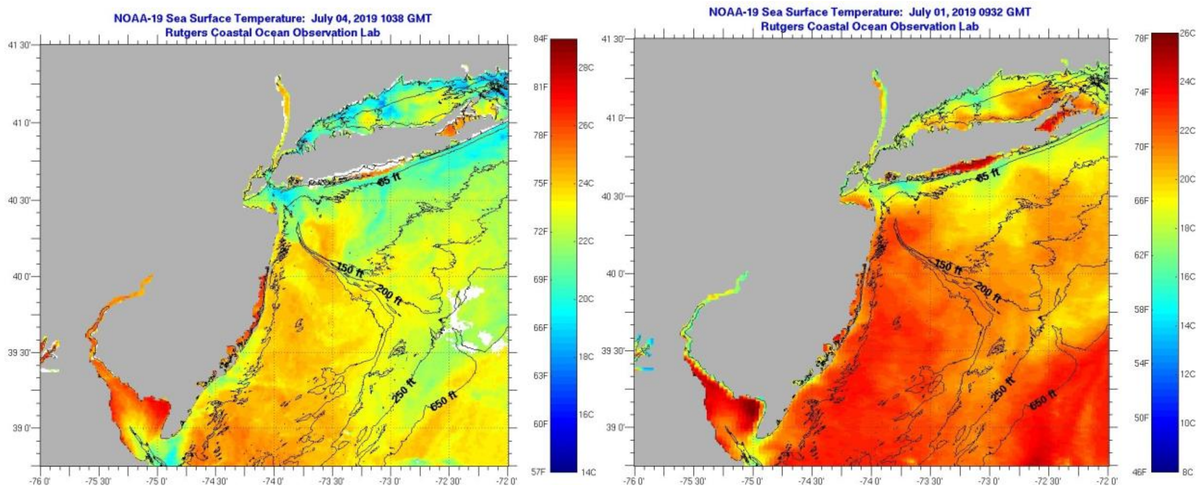

Figure S2. Sea Surface Temperature on July 04 2019 (3 LT) (left) and on July 01 2019 (4 LT) (right). The images were archived on the website of The Coastal Ocean Observation Lab of Rutgers University (https://marine.rutgers.edu/cool//sat_data/?nothumbs=0&product=sst&region=sw06)

3. Borough-based GAMs model

By statistics, Staten Island has the highest tree cover among boroughs with almost 30 %, followed by The Bronx (23 %), Manhattan (20 %), Queens (18 %), and Brooklyn (16 %). The distribution of building heights among these boroughs demonstrated by the Building Height dataset. A series of borough-level GAMs were conducted separately for each borough, as summarized in Figure S3 - S8.

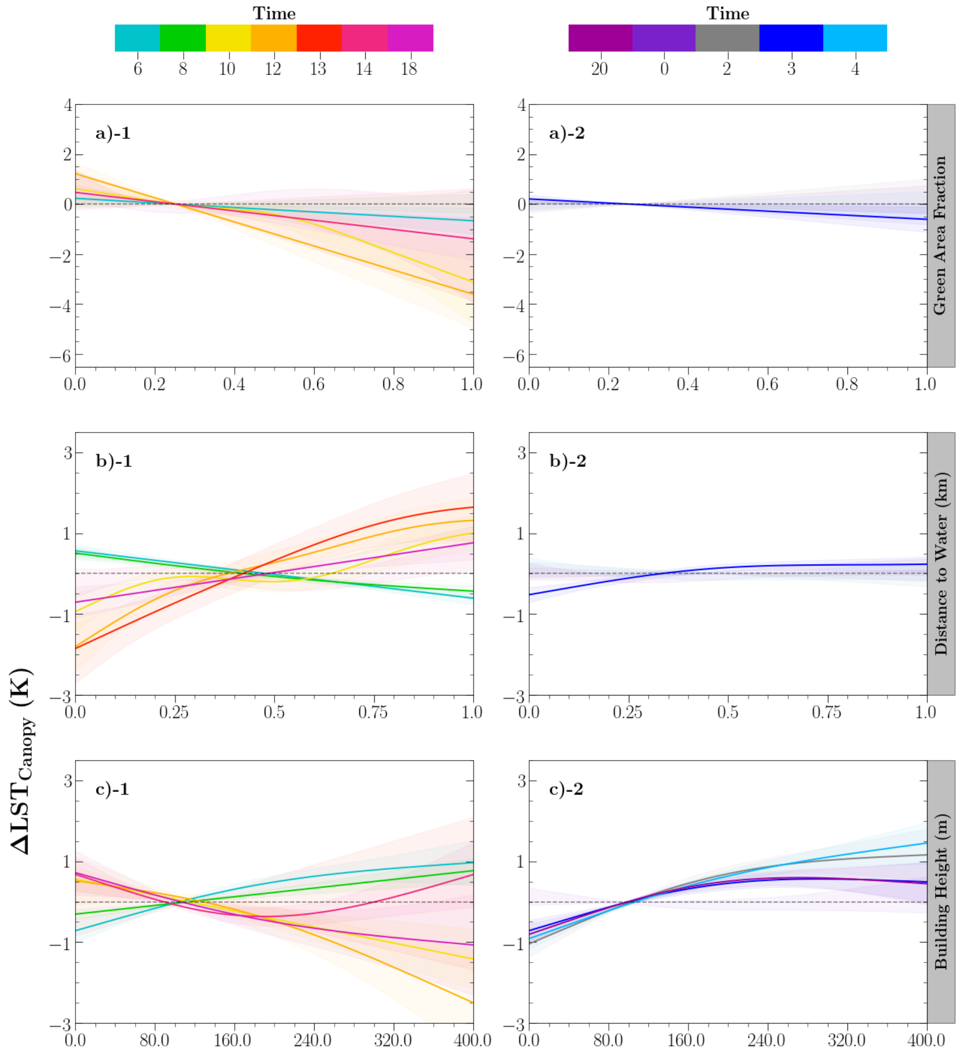

Figure S3. Estimated effects of urban greenspaces, distance to waterbodies, and building height on the spatial and diurnal patterns of $\Delta\, LST_{Canopy}$ for **Manhattan**. The left column is for daytime hours and the right column is for nighttime hours. Domain mean LST was used as a reference value for $\Delta\, LST_{Canopy}$. $\Delta\, LST_{Canopy}$ equals zero is indicated as black dashed lines. The shaded regions illustrate the confidence interval 95% of GAMs fitting.

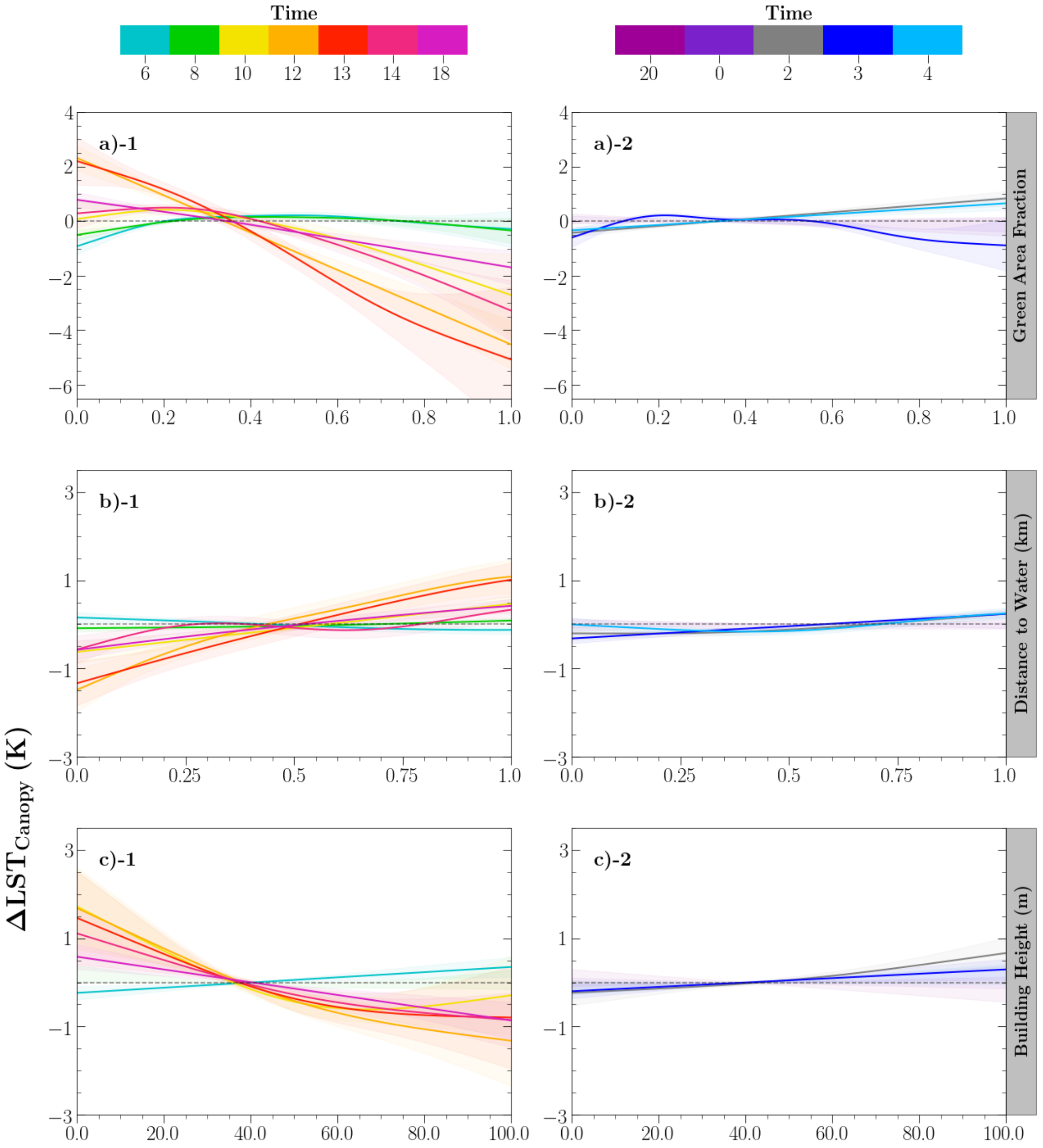

Figure S4. Estimated effects of urban greenspaces, distance to waterbodies, and building height on the spatial and diurnal patterns of $\Delta\, LST_{Canopy}$ for **The Bronx**. The left column is for daytime hours and the right column is for nighttime hours. Domain mean LST was used as a reference value for $\Delta\, LST_{Canopy}$. $\Delta\, LST_{Canopy}$ equals zero is indicated as black dashed lines. The shaded regions illustrate the confidence interval 95% of GAMs fitting.

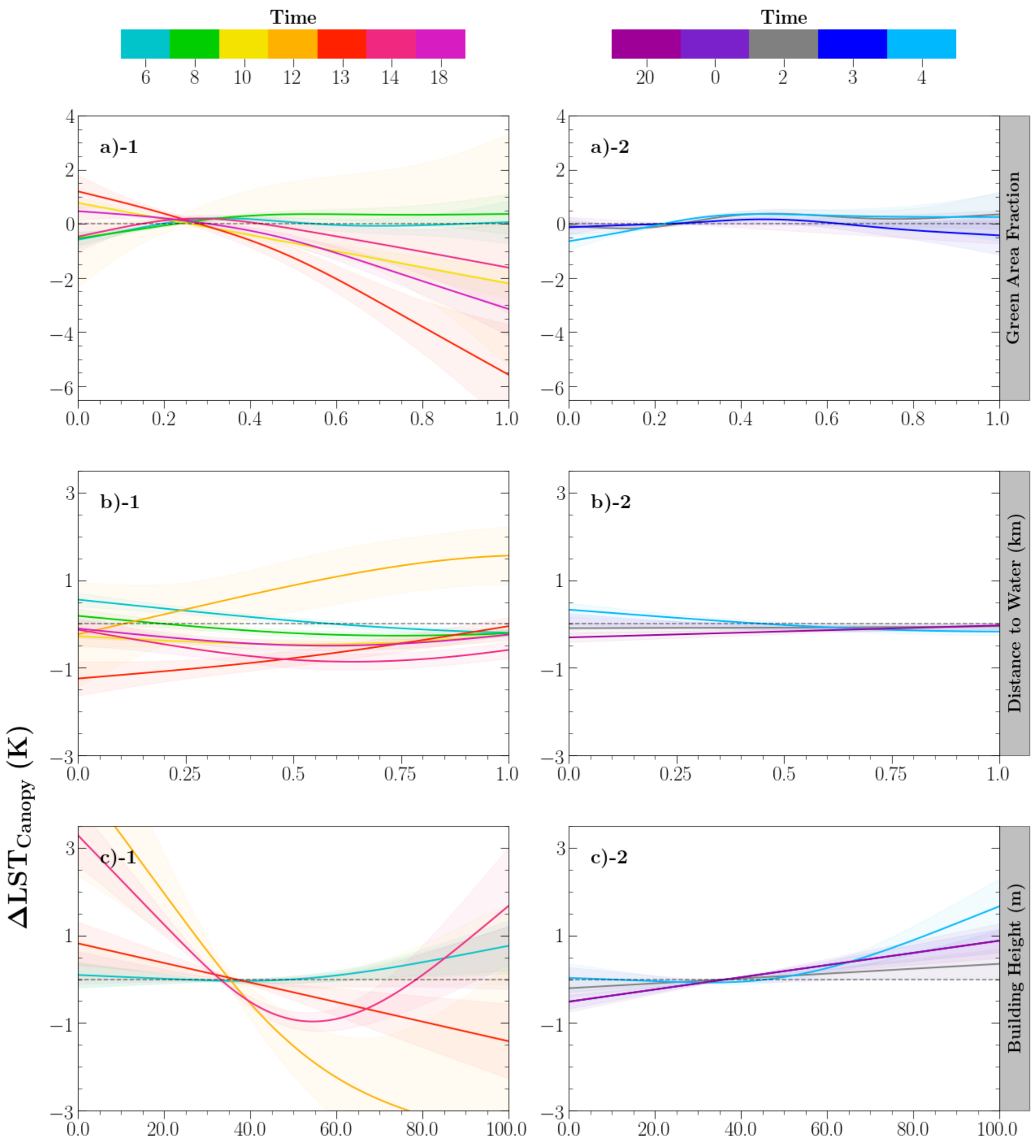

Figure S5. Estimated effects of urban greenspaces, distance to waterbodies, and building height on the spatial and diurnal patterns of $\Delta\, LST_{Canopy}$ for **Brooklyn**. The left column is for daytime hours and the right column is for nighttime hours. Domain mean LST was used as a reference value for $\Delta\, LST_{Canopy}$. $\Delta\, LST_{Canopy}$ equals zero is indicated as black dashed lines. The shaded regions illustrate the confidence interval 95% of GAMs fitting.

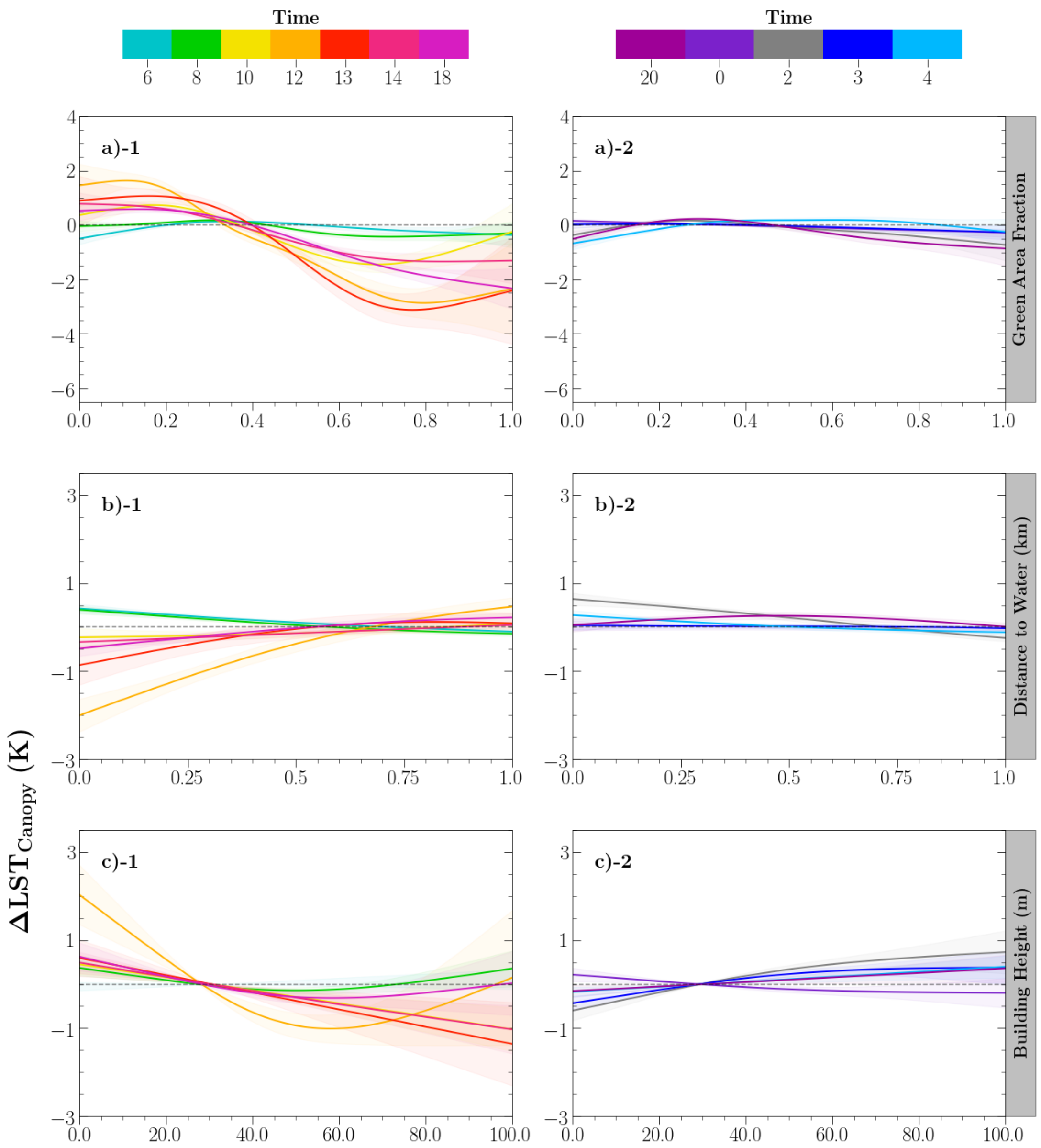

Figure S6. Estimated effects of urban greenspaces, distance to waterbodies, and building height on the spatial and diurnal patterns of $\Delta\, LST_{Canopy}$ for **Queens**. The left column is for daytime hours and the right column is for nighttime hours. Domain mean LST was used as a reference value for $\Delta\, LST_{Canopy}$. $\Delta\, LST_{Canopy}$ equals zero is indicated as black dashed lines. The shaded regions illustrate the confidence interval 95% of GAMs fitting.

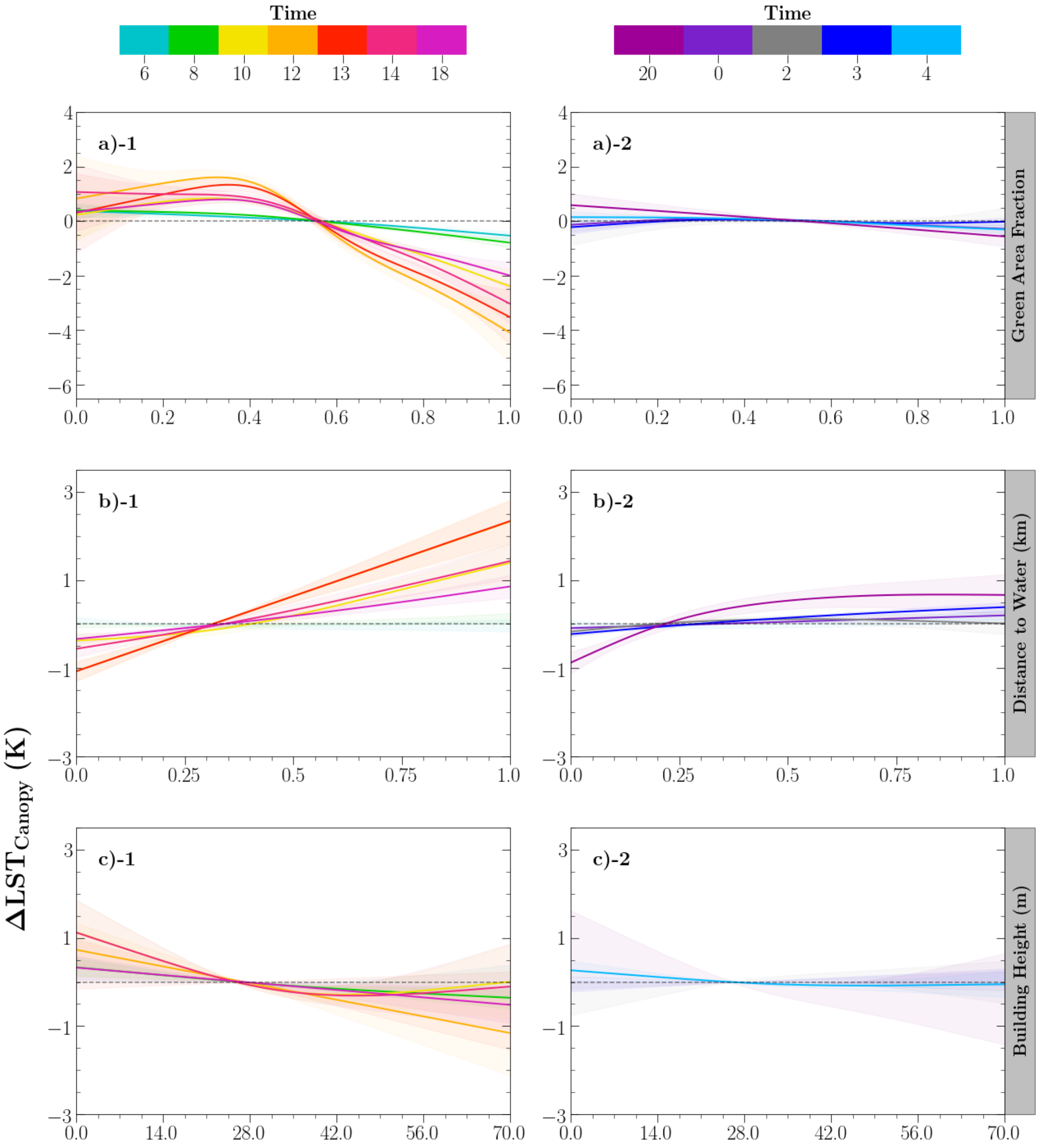

Figure S7. Estimated effects of urban greenspaces, distance to waterbodies, and building height on the spatial and diurnal patterns of $\Delta\, LST_{Canopy}$ for **Staten Island**. The left column is for daytime hours and the right column is for nighttime hours. Domain mean LST was used as a reference value for $\Delta\, LST_{Canopy}$. $\Delta\, LST_{Canopy}$ equals zero is indicated as black dashed lines. The shaded regions illustrate the confidence interval 95% of GAMs fitting.

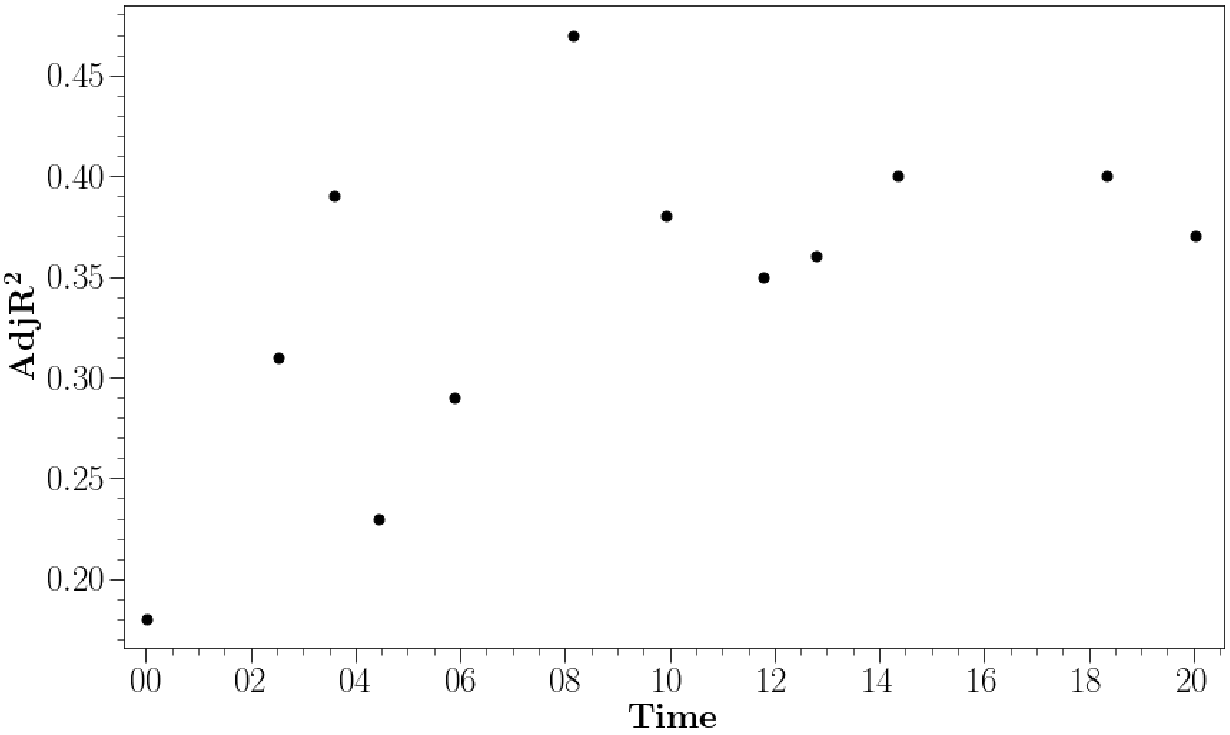

Figure S8. Adjusted R^2 of the GAMs model from the city scale analysis.

4. **Sensitivity test to decide an appropriate kernel size for End-member Temperature Estimation**

In order to decide an appropriate kernel size for End-member Temperature Estimation, we conducted a sensitivity analysis for various combinations of moving windows. There are 3 sets of moving size were chosen to test the sensitivity of this approach on estimating canopy temperature. As concluded, we found that the distribution of canopy temperature using these moving window sets is not significantly different. Therefore, we chose set [10,5], which means moving window size of 10 x 10 pixels and moving steps of 5 pixels or in other words, kernel size of 5 x 5 pixels. Based on this decision, we assumed that the end-member temperatures are homogeneous within a neighbor scale of 5 x 5 pixels (or, 350 x 350 meters).

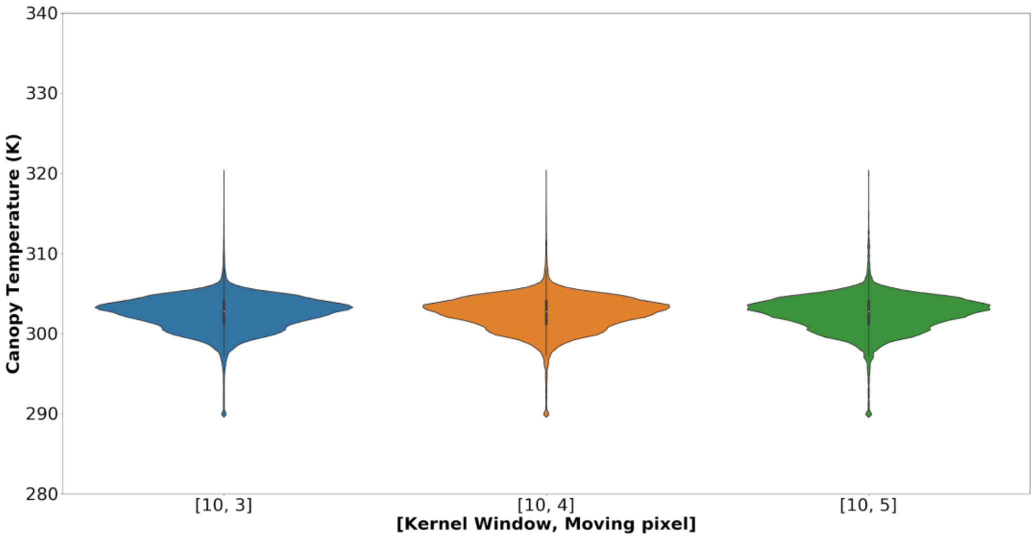

Figure S9. The distribution of estimated canopy temperature is plotted for various window sizes and moving pixels. The x-axis represents the symbol for each test, for example, [10,5] means window size 10 x 10 pixels with a moving step of 5 pixels.

5. **Model performance of End-member Temperature Estimation approach**

In our analysis, we conducted an MLR method to statistically decompose the sub-pixel temperatures among different urban elements at each pixel using Equation 1 in the main document. To confirm the consistency of the results, we showed the distribution of the residuals extracted from the model output for two times of the day: at 13 Local Time (LT) and at 3 LT (Figure S10)

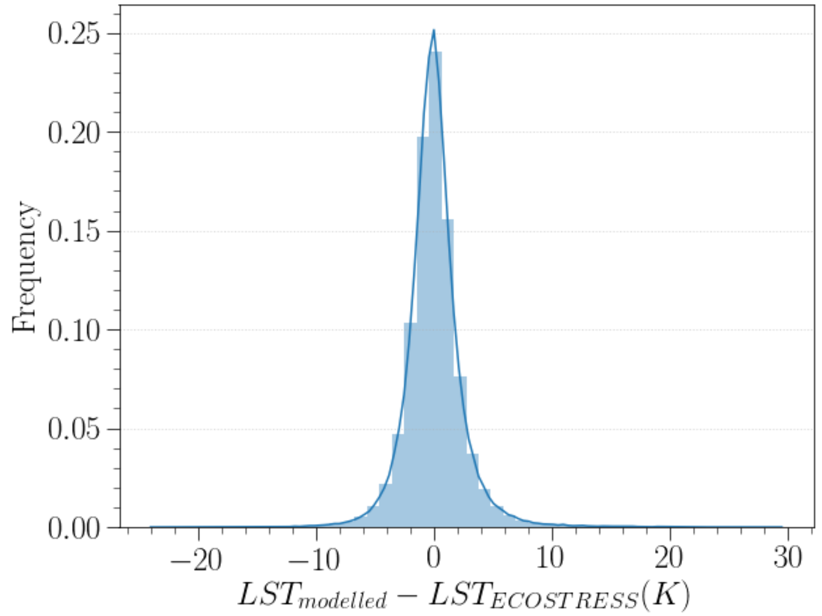

a) Daytime (13 LT)

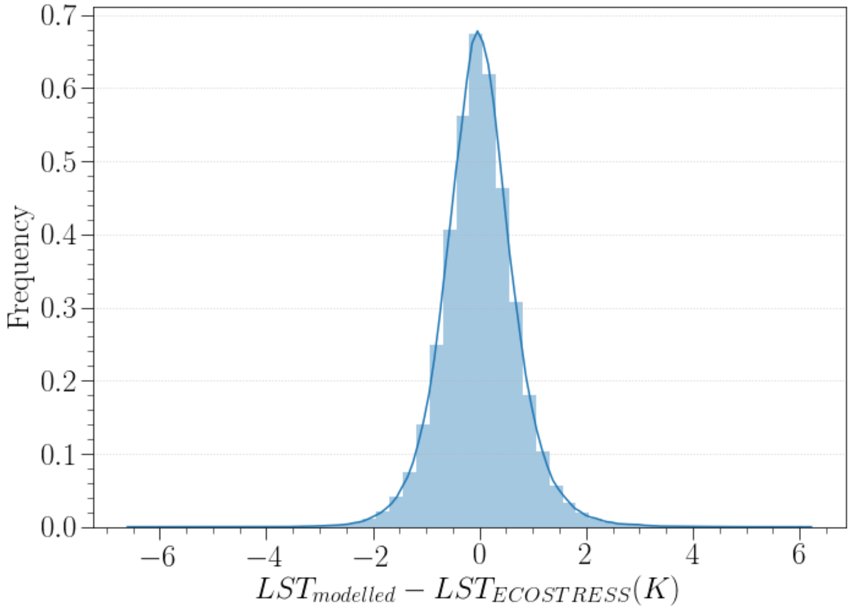

b) Nighttime (3 LT)

Figure S10. Histogram of residuals from the End-member surface temperature estimation (a) Daytime (13 LT) and (b) Nighttime (3 LT). The residuals have Gaussian distribution that confirms the reliability of the least-squares solution using constrained rules for sets of MLR equations.

Besides, we also showed residual maps (the difference between modeled LST and ECOSTRESS LST) for those 2 days in Figure S11. It could be seen that overall, our model has acceptable performance, proven by the fact that the residual values follow Gaussian distribution (Figure S10) and the residual maps show near homogenous residual values in the range of -2 to 2 (K). Despite some outliers (e.g., high absolute residual values), especially on the daytime residual map (Figure S11 a), our modeled LST values show reliable results compared to the ECOSTRESS LST, which confirms a good accuracy in our downscaled approach.

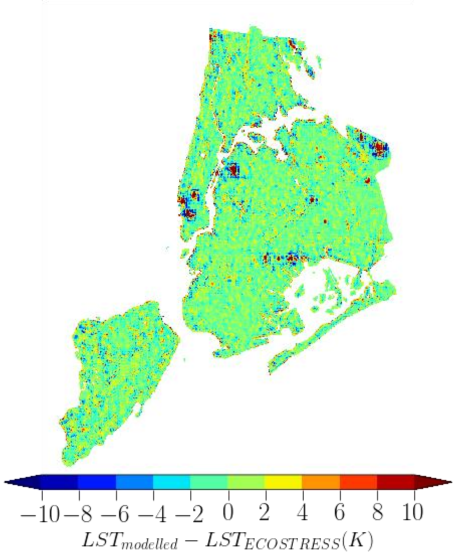

(a) Daytime (13 LT)

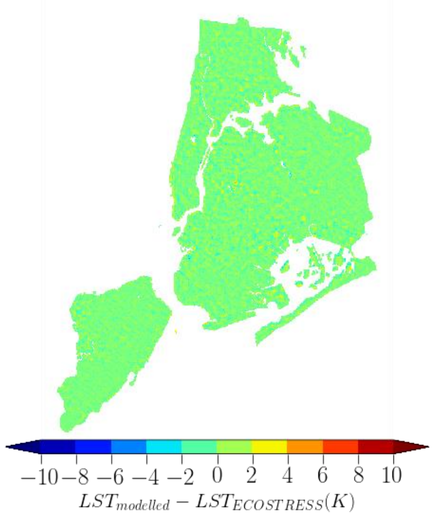

(b) Nighttime (3 LT)

Figure S11. Residual maps (the difference of modeled LST and ECOSTRESS LST) (a) Daytime (13 LT) and (b) Nighttime (3 LT). Maps created in Python 3, using the canopy temperature dataset

generated based on the downscaled approach and ECOSTRESS LST dataset (link is provided in the Data Availability section).
